# Aging Positively: Redefining Resilience & Legacy With HIV

**DOI:** 10.1093/geroni/igaf122.003

**Published:** 2025-12-31

**Authors:** Haley Sanner

**Affiliations:** Colorado Health Network, Denver, Colorado, United States

## Abstract

Founded during the AIDS epidemic, Colorado Health Network (CHN) is a statewide organization providing services for people living with HIV (PLWH). The advent of antiretroviral therapy (ART) drastically increased life expectancy, thus presenting unique quality of life concerns for older PLWH (over 50% of clients), especially long-term survivors. In addition to the physical health complexities, older PLWH, especially long-term survivors, experience higher rates of depression, loneliness, and fractured support networks. Additionally, HIV is an intersectional disease that disproportionately affects people of color, LGBTQ+ people, low-income people, people experiencing homelessness, and people with disabilities. CHN established Healthy Aging Programs (HAP) to prioritize PCTI programming to improve physical and mental health outcomes for older PLWH. In collaboration with CHN Behavioral Health and Peer Leaders, HAP has developed a robust ‘Aging Positively’ psychosocial program to address the intersection of ageism, HIV-stigma and social isolation. The original 6-week curriculum explores themes of themes of positive aging pertaining to older PLWH - including resilience, self-compassion, survivorship, stigma, and intersectionality. In collaboration with older PLWH, a follow-up offerings that commemorates the lived experiences and legacies of older PLWH by employing storytelling, creative aging and intergenerational methods. By integrating equitable approaches to program design, CHN shifts the perspective from ‘If we build it, will they come?’ to ‘If they build it, will they use it?’ to intentionally co-create programming with older participants. This session will highlight implementation science methods, psychosocial contexts, structural elements, cross-departmental collaboration, community engagement approaches and lessons learned from this innovative program.

